# Toxic Effects of Single Antibiotics and Antibiotics in Combination on Germination and Growth of *Sinapis alba* L.

**DOI:** 10.3390/plants9010107

**Published:** 2020-01-15

**Authors:** Ulrike Timmerer, Lennart Lehmann, Ewald Schnug, Elke Bloem

**Affiliations:** Julius Kühn-Institut, Federal Research Centre for Cultivated Plants, Institute for Crop and Soil Science, Bundesallee 69, 38116 Braunschweig, Germany

**Keywords:** combinatory effects, copper (Cu), enrofloxacin, phytotoxicity, *Sinapis alba* L., sulfadiazine, tetracycline

## Abstract

Antibiotics enter agro-ecosystems via the application of farmyard manure, sewage sludge, animal by-products, or digestates. There are many open questions regarding the behavior of such compounds in the soil like their adsorption, degradation, half-life, and their effects on soil organisms and plants. The impact of antibiotics on the development of antibiotic resistance genes in the environment is regarded as the most important effect that endangers the environment as well as human health. Nevertheless, direct plant toxicity, especially of different antibiotics and heavy metals at the same time, can be of importance as well. In the current study, commercially available phytotoxkits were tested with regard to the toxicity of single antibiotics and antibiotics in combination with the root growth of *Sinapis alba* L. Additionally, a pot trial was conducted to study the transfer of the observed phytotoxkits results in more complex systems. The phytotoxkits revealed direct toxicity of antibiotics on root development only at high concentrations. The highest toxicity was determined for sulfadiazine, followed by tetracycline and enrofloxacin, showing the least toxicity. When two antibiotics were tested at the same time in the phytotoxkit, synergistic effects were detected. The pot trial indicated lower effect concentrations for enrofloxacin than determined in the phytotoxkit and, therefore, to higher toxicity on plant growth.

## 1. Introduction

The common agricultural fertilizer practice by applying farmyard manures, sewage sludge, animal by-products, digestates, or other organic nutrient sources to the field always pose a risk to deliver contaminants to the soil such as antibiotics, heavy metals, or pathogens beside valuable nutrients and organic matter [1]. Very often, the toxicity of new rising nutrient sources is not sufficiently evaluated. Therefore, it is hardly possible to get information on the environmental risks when applying fertilizer sources like digestates or recycling materials to agricultural fields. The contamination of animal manures with antibiotics and their metabolites is of increasing relevance. The contamination is caused by the intensive use of antibiotics in livestock farming, with nearly 65,000 t per year worldwide [2]. Additionally, via sewage sludge application, very different organic and inorganic contaminants, as well as pathogens, enter the soil system. Only a small proportion of the antibiotics that were administered, to farm animals remain in the animals or were metabolized, while a great portion of up to 90% is excreted either with the urine or feces [3]. Grazing livestock will directly contaminate the meadow when treated with antibiotics, while in case of indoor housing of animals administered antibiotics will enter the soil via slurry, digestate, or farmyard manure application. Antibiotics are designed to affect microorganisms. Therefore, it is very likely that antibiotics released into the environment will have an effect on the soil microbiome. The different classes of antibiotics show a diverse environmental persistence in the soil ranging from a few to several hundreds of days [4].

It is important to characterize the toxicity of contaminants separately, but it is even more important to investigate the toxicity of different compounds in combination, as a mixture of antibiotics together with heavy metals and other organic contaminants is present in most of the organic nutrient sources. The toxicity of a substrate can be analyzed without knowing, which parameter is responsible for the effect [5] or special toxkits can be used to analyze the effect of selected compounds either alone or in combination with each other. With regard to antibiotics, there are two different important aspects when analyzing the eco-toxicity: (1) the risk of the formation and spread of antibiotic resistance genes (ARGs) in soil microorganisms and (2) the acute toxicity on the microbial community in the soil, on other soil organisms like earthworms and on plant growth and development.

In the present study, the acute toxicity of antibiotics on root development and plant growth parameters was studied. It is important to mention that the development of ARGs can be promoted by much lower concentrations than the ones, which are necessary to cause toxic effects on plant growth. For example, tetracycline (TC) concentrations of 0.1–1 mg/kg in liquid manure were shown to cause a significantly increased gene transfer of transposon-based TC resistance [6], a concentration often detected in agricultural soils adjacent to feedlots in China [7]. ARGs were also detected in non-manured soils, indicating the ubiquitous occurrence of them in agricultural soils [8]. Generally, repeated manure application provides positive selection pressure for bacteria that are resistant to special antibiotics, resulting in an accumulation of ARGs in agricultural soils [8,9,10]. Different heavy metals, like Hg, Cu, and Zn accelerated the selection pressure for ARGs and have the potential to increase ARG abundance [7].

Much higher antibiotic concentrations are necessary to cause direct toxicity on soil organisms and plants: for example, contamination of 10 mg enrofloxacin/kg soil over eight weeks was necessary to cause oxidative stress and a reduced burrowing activity in the earthworm *Eisenia fetida* [11]. Michelini et al. [12] observed that a concentration of 10 mg sulfadiazine/kg soil exhibited an adverse effect on root growth of willow (*Salix fragilis* L.) and maize (*Zea mays* L.) over an exposure time of 40 days but no effect on the aboveground biomass yield was measured. In contrast, contamination with 200 mg sulfadiazine/kg soil, which is high above the concentrations that can be expected in agricultural soils, caused a clear biomass reduction and exhibited a toxic effect on plant development [12].

The aim of the present study was the evaluation of the toxicity of antibiotics from different classes (tetracyclines (TCs), sulfonamides (SAs), and fluoroquinolones (FCs)) on germination and growth of *Sinapis alba* L. seeds. These three classes of antibiotics are commonly used in veterinary and human medicine, with TCs having a share of 30% of the antibiotics used in Germany in 2011 [13] and about 66% of that used in Europe [14]. SAs are used to a lower degree, with consumption rates of up to 23% in Germany [14] and 10% in the EU in 2012 [15]. In Germany, recent data show a decrease in TC and SA consumption in animal husbandry but an increasing usage of FCs [16]. FCs are used with a growing proportion in the last years because of their higher activity and were detected quite frequently in farm-derived fertilizers [13,16].

Under natural conditions, organisms are not exposed to single toxicants, but to several compounds at the same time, that can affect each other. The investigation of the eco-toxicity of contaminants can help to understand the behavior of the compounds in the environment and deliver information on its bioavailability. Chemical data alone can over- or underestimate the toxicity of a compound under environmental conditions as different compounds can show additive, antagonistic, or synergistic effects, and the toxicity is affected by environmental factors as well [5]. A number of tests were designed for aquatic systems, and only a few tests are commercially available to characterize the toxicity in the soil system and on plant growth. In the present study, a phytotoxkit for seed germination and early root development was used to screen different single compounds, as well as combinations of antibiotics for their toxicity. Additionally, a pot trial was conducted to unravel if the easy to obtain phytotoxkit results can be transferred to the more complex system of a pot trial. Typical key values for toxicity were identified by eco-toxicity tests that help to classify the toxicity of a xenobiotic like the LC_50_ value, which is the concentration where 50% of the organisms are dead (LC-lethal concentration) or the EC_50_ value (EC-effect concentration), the concentration where 50% of the organisms show an effect like a lower root growth [5]. Opportunities and drawbacks of such easy-to-perform tests are highlighted in the present work.

## 2. Results

### 2.1. Toxicity of Antibiotics on Germination of Sinapis alba L. Seeds Observed by Phytotoxkits

*Sinapis alba* L. was chosen for the phytotoxicity test because of its agronomic relevance and uniformly growth in pre-trials. The test was performed with different antibiotics (Enrofloxacin EN, Sulfadiazine SD, Tetracycline TC) and copper (Cu). Initially, the effect of each compound alone was tested, and afterward combinations were analyzed based on the results of single compounds to test for combinatory effects.

**Figure 1:**
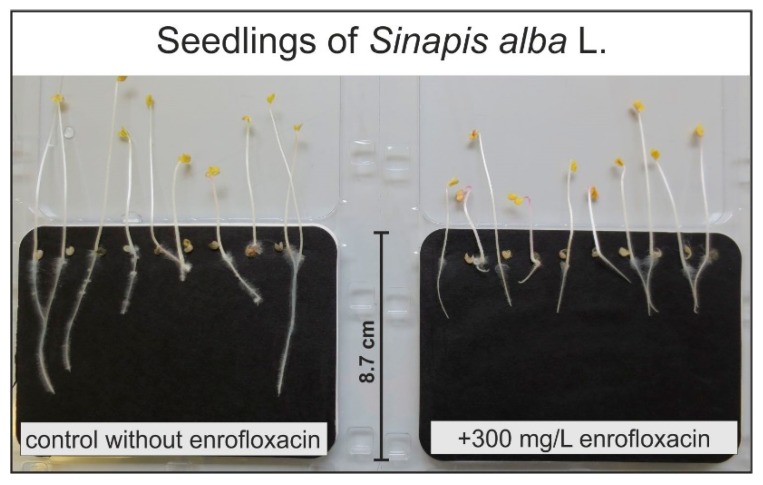
Effect of enrofloxacin application in a citric acid buffer on root growth of *Sinapis alba* L. after 120 h of treatment, (phytotoxkit (MicroBioTests Inc. Gent, Belgium) performed in 0.5 g/L citric acid at pH 6).

Seeds were exposed to the different test solutions, and after 120 h, germination and root length were monitored in relation to control seeds, which germinated on the pure buffer solution (citric acid buffer at pH 6; for experimental conditions see Section 4.1). Exemplary in Figure 1, a phytotoxicity test is shown for *Sinapis alba* L., where root growth in a control treatment is compared to one at the highest EN level.

**Figure 2:**
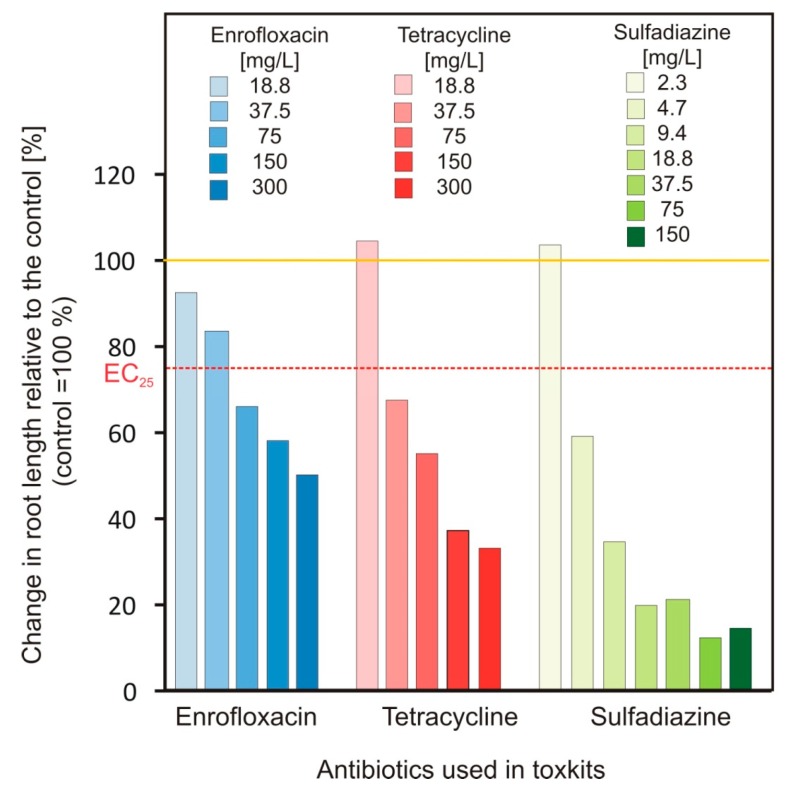
Relative change in root length of *Sinapis alba* L. seedlings treated with increasing concentrations of different antibiotics observed in phytotoxkits (MicroBioTests Inc. Gent, Belgium; tests performed in 0.5 g/L citric acid buffer at pH 6 for 120 h).

After 120 h of treatment an effect of all three investigated antibiotics on root length was observed (Figure 2), but different concentrations proved to be effective. 300 mg/L of EN was necessary to reduce the root length of the seedlings by 50%, while at the same dose of TC (300 mg/L) root length was reduced by more than 60%. Seedlings reacted most sensitive to SD, where at a dose of 150 mg/L SD root length was already reduced by more than 80% (Figure 2). At the lowest tested concentration of TC (18.8 mg/L) and SD (2.3 mg/L), the root growth was slightly increased in comparison to the control, which indicates activation of root growth at low antibiotic concentrations.

Based on the phytotoxkit results, effect concentrations were calculated for the different test compounds, and different key values (EC_25,50,70_) are compiled in Table 1 for the single compound tests. The highest sensitivity of *Sinapis alba* L. seeds was determined for SD, where root growth was reduced by 25% in relation to the control at a concentration of 2.7 mg/L. The toxicity of TC and EN on the root growth of *Sinapis alba* L. was in a similar range, and about 15-times lower than that of SD. The EC_70_ values (Table 2) reveal that EN is less toxic than TC at higher concentrations because a 3.8-times higher EN concentration was necessary to reduce the root length by 70%.

No toxicity was observed for copper (Cu) in the concentration range of 0.5–24 mg Cu/L that was tested in the phytotoxkit. It is possible that the test duration of 120 h was too short to trigger a toxic effect or that much higher concentrations are necessary to reduce germination and early root growth.

**Figure 3:**
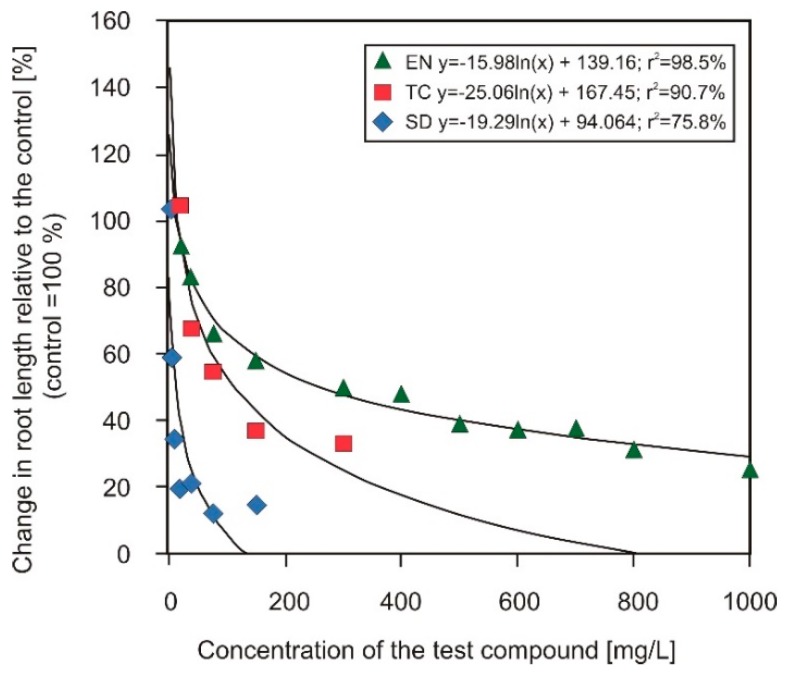
Regression curve of the change in root length growth of *Sinapis alba* L. in relation to the concentration of enrofloxacin (EN), tetracycline (TC) and sulfadiazine (SD) in the phytotoxkits after 120 h of treatment (MicroBioTests Inc. Gent, Belgium; tests performed in citric acid buffer (0.5 g/L citric acid) at pH 6).

In a second step, the different antibiotics were combined to investigate possible combinatory effects. For this purpose, one antibiotic was applied in a fixed concentration, while the second one was applied in the same variable concentrations as in the test before. The fixed concentration was chosen based on the previous trial. Fixed SD and TC concentrations were chosen close to the EC_25_ value. In the case of EN, a slightly higher value was chosen (about EC_40_) because the root length was less affected by the higher concentrations, and the curve progression flattened with higher concentrations (Figure 3).

In Table 2, the results of the combinatory toxicity of EN, TC, and SD are presented, and the EC_70_ values are shown for the single compounds and for the combinations. For example, a concentration of 926 mg/L EN was necessary to reduce root growth by 70% in comparison to the control. When at the same time, 3 mg/L SD was present, only 167 mg/L EN was necessary to obtain the same reduction.

**Figure 4:**
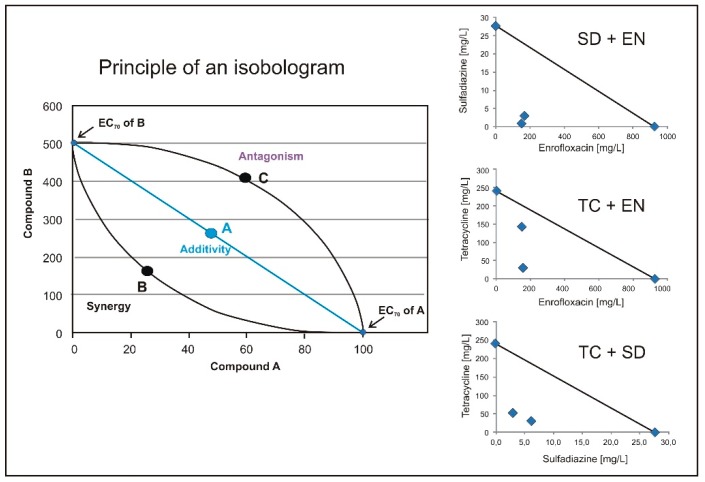
Principle of an isobologram for an effect concentration of 70% (EC_70_): dots on line A will result when the effect of the two compounds is additive. Measured values below the line B indicate a synergistic and values above C to an antagonistic effect [17]. On the right hand side, the EC_70_ isobolograms for the combination of sulfadiazine (SD) and enrofloxacin (EN), and that of tetracycline (TC) and enrofloxacin (EN) and of tetracycline (TC) and sulfadiazine (SD) are shown.

All EC_70_ values decreased when more than one antibiotic was present in the test solution at the same time, indicating increasing toxicity (Table 2). From the data alone, it is hardly possible to decide if the effect is additive or synergistic. Isobolograms can be calculated [17] from the observed data and can be used to distinguish if the effect between two compounds is additive, antagonistic, or synergistic (Figure 4).

From Figure 4, it is obvious that all EC_70_ values for the combinations of the tested antibiotics are well below the additive line. Therefore, all three antibiotics show synergistic effects when they were available in the phytotoxkits with *Sinapis alba* L. at the same time.

In addition, the combination of Cu with a fixed EN concentration was tested, but again, it was not possible to determine any changes in growth in relation to Cu, which is why no combination is shown for Cu. It seems to be one major limitation of the phytotoxkits, that the duration of the experiment is too short for elements such as Cu to unfold a measurable effect on plant development. Therefore, an additional pot experiment was performed to investigate the effect of increasing EN concentrations in combination with Cu on plant growth of *Sinapis alba* L. over a longer period of time (28 days) until the start of flowering.

### 2.2. Toxicity of Enrofloxacin (EN) and Copper (Cu) on Growth of Sinapis alba L.

A sand culture experiment was conducted to study the reliability of the phytotoxkit results. The experimental setup is described under Section 4.2. The phytotoxkit results reflect the effects of a compound on germination and early root growth while in the pot trial, the whole vegetation cycle can be analyzed. To get an idea if the phytotoxkit results are of relevance also for further crop growth, it is important to analyze if such results can be transferred to vegetation trials. *Sinapis alba* L. was grown until the main growth, and the effect of EN and Cu alone and in combination was evaluated.

**Figure 5:**
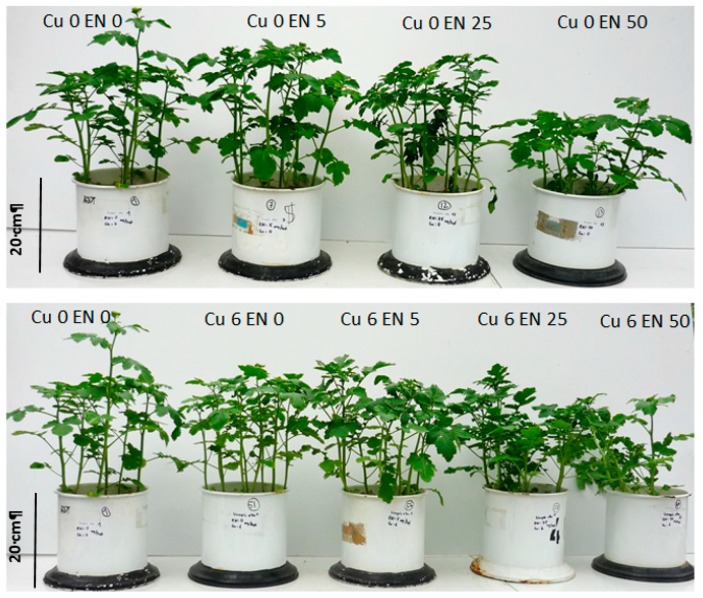
Biomass production of *Sinapis alba* L. in relation to increasing enrofloxacin concentrations with and without copper application. (*Sinapis alba* L. seeds were grown for 28 days in Mitcherlich pots containing 7 kg of sand, fertilized with all necessary nutrients. Enrofloxacin (EN) was applied before sowing at increasing concentrations: 0, 5, 25, 50 mg/pot EN without and in combination with 6 mg/pot copper (Cu).

**Figure 6:**
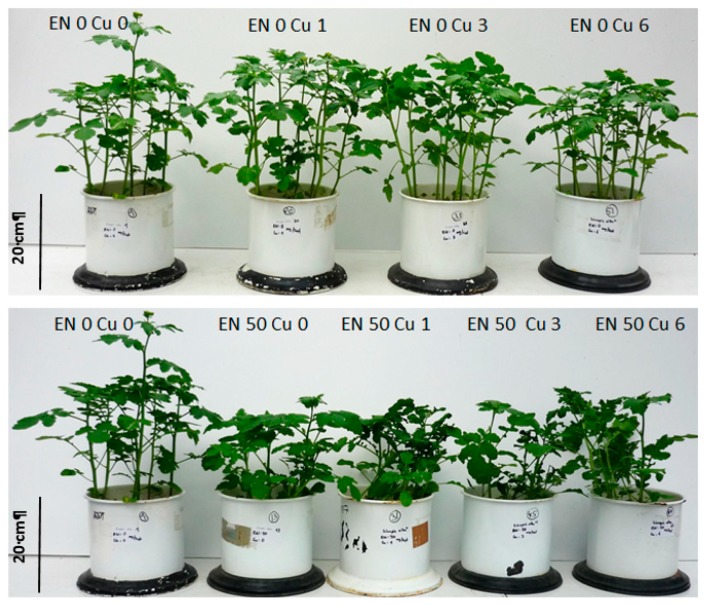
Biomass production of *Sinapis alba* L. in relation to increasing copper concentrations with and without additional enrofloxacin application. (*Sinapis alba* L. seeds were grown for 28 days in Mitcherlich pots containing 7 kg of sand fertilized with all necessary nutrients. Copper (Cu) was applied before sowing at increasing concentrations: 0, 1, 3, 6 mg/pot Cu without and in combination with 50 mg/pot enrofloxacin (EN).

EN only reduced the growth of *Sinapis alba* L. significantly when addressed at very high concentrations (Figure 5), while Cu application caused no yield reduction at the tested concentrations and did not raise EN toxicity (Figure 5 and Figure 6).

In Table 3, fresh weight (FW) and dry weight (DW) biomass data of the pot trial are shown. The Cu application did not impair root or vegetative growth. Root fresh weight (FW) was significantly lower at a concentration of 25 and 50 mg per pot EN. On a dry weight basis, this effect was only significant at the highest dose of 50 mg per pot EN. Vegetative growth was significantly reduced only at the highest dose of 50 mg EN per pot, which is equivalent to 7.1 mg EN/kg soil. An interaction between Cu and EN was determined for root fresh weight production—with increasing Cu concentration, the toxicity of EN seemed to increase. Therefore, these data indicate an interaction between Cu and EN that cannot be observed visually or in dry matter production.

The Cu uptake of the plants was not affected by the EN concentration only by the Cu application, to the soil (Table 4). With increasing Cu application the Cu content in leaves, stems, and roots increased, and this effect was significant when 3 mg or more were applied to the pots. The highest Cu content was determined in the roots followed by leaves, and the lowest content was determined in the stems.

Also, EN was determined in the plant material. Initially, representative samples were analyzed for EN because of the time-consuming and expensive extraction procedure and determination. As the obtained data did not indicate an effect of Cu application on EN uptake, only the effect of EN application on the EN content in the plant material was analyzed. Control plants were also not analyzed because no EN could be expected in these samples. Therefore, no ANOVA was calculated for EN in Table 5. Only trace amounts of EN could be determined in the plant material compared to the high application rates.

The data in Table 5 show that EN was taken up by the plants in a concentration-dependent way. The highest concentrations were observed in the roots. The fact that EN could be detected in the aerial plant parts clearly indicates to uptake of EN from the soil and to transport within the plant. Nevertheless, the proportion, which was taken up by the plants, was very low and accounted for 0.001–0.017% of the amount that was applied to the pots. In comparison, the copper uptake accounted for 4–12% of the total application to the pot. The translocation factor (Tf) is a good measure to get an impression of how much EN was translocated from the roots to the shoots. At the lower EN doses, a higher proportion of about 69 and 83% of EN taken up is transported to the aerial part, which is a substantial proportion. At the highest dose of 50 mg/pot, EN 51% was translocated to aerial parts, indicating a higher proportion bond to the roots.

EN seems to be quickly and strongly bond to the soil matrix, which was indicated by the suction water and soil analysis. The suction water analysis revealed that the amount of EN in the soil water was below the limit of quantification and only at the first sampling five days after starting the experiment it was possible to detect traces of EN in the suction water.

**Figure 7:**
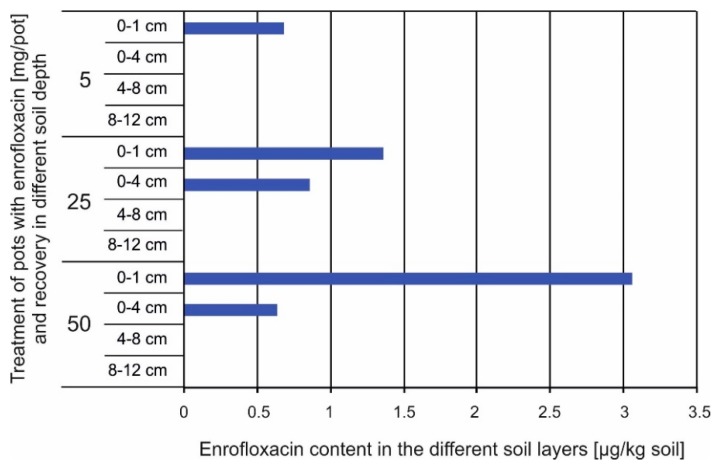
Enrofloxacin (EN) bound to the sandy soil matrix in different soil depth in a Mitscherlich pot 28 days after applying 5, 25, or 50 mg EN per pot. (Extraction was performed from the additional pots containing 7 kg of sand without vegetation with methanol containing 1% formic acid; no statistics could be revealed as repetitions were only made from the planted pots).

In addition, the soil analysis revealed only traces of the applied EN (Figure 7). Soil extraction using methanol (containing 1% formic acid) was not strong enough to extract substantial amounts of EN from the soil. Traces of EN could be detected only close to the soil surface and up to 4 cm in depth but not deeper, indicating a fast and strong sorption of EN to the soil matrix. Soil analysis at the highest EN level (50 mg EN per pot) revealed that also traces of ciprofloxacin below the limit of quantification could be detected, which is a degradation product from EN. This indicates degradation of EN during the experimental time.

## 3. Discussion

The direct phytotoxicity of antibiotics on plant growth can be estimated by using phytotoxkits but needs to be proven by plant growth experiments. In the conducted trials, very high concentrations of antibiotics were tested for their toxicity on plant growth to evaluate the effect concentrations (EC_25_, EC_50_, and EC_70_) as key data for toxicity. Even if such high contaminations were rarely found in manure samples or soils, they could be of relevance. High antibiotic contaminations were reported, for example, for the synthetic fluoroquinolones, which are used for the therapy of bacterial infections in humans and animals and which belong to the most potent antibacterials in veterinary medicine [18]. In China, antibiotics of the class of fluoroquinolones can be detected in high frequency in reclaimed water and groundwater in the ng/L range [19]. Therefore, special emphasis was given in the present study to enrofloxacin (EN), one antibiotic out of the class of the fluoroquinolones. For example, norfloxacin and ciprofloxacin were detected in concentrations of 6.2–9.8 mg/kg and 3.0–5.8 mg/kg, respectively, in natural soil samples [20]. Such high concentrations are comparable to the ones used in the experiment of the present study. Effluents of drug manufactures can also show high concentrations of antibiotics. A maximum concentration of 31 mg/L ciprofloxacin was detected in sewage in India [21]. This is a concentration, where toxic effects on plant growth can be expected according to our results. Moreover, it needs to be stressed again that organic nutrient amendments and sewage always contain a lot of different potentially toxic compounds that can cause combinatory effects. The present work indicates that it is a complex task to get reasonable and reliable data on the combinatory effects of different toxic compounds on plant growth but that it is worth to study them.

### 3.1. Toxicity of Antibiotics on Plant Germination and Early Plant Development

The data of the here presented phytotoxkits clearly revealed that the different antibiotics unfold very different toxicity on early root growth of *Sinapis alba* L. Important toxicity values are the effect concentrations (EC values), which can be calculated for different reduction rates. EC_25_ values of 2.7 mg/L for SD, 40 mg/L for TC, and 55 mg/L for EN were determined for early root development in the present study by a commercially available phytotoxkit. Comparable results were reported by Eguchi et al. [22], who determined an EC_50_ value of 2.2 mg/L SD for the growth of *Selenastrum capricornutum* (Printz) in liquid media. According to Liu et al. [23], a root growth reduction by 50% was obtained when the nutrient solution contained 69 mg/L TC in the case of *Oryza sativa* L., 203 mg/L TC in case of *Cucumis sativus* L., and 57 mg/L in case of *Cichorium endivia* L. In the current study, the EC_50_ at 109 mg/L TC for *Sinapis alba* L. was well within the range of the reported data. In the case of *Lactuca sativa* L., a much higher EC_50_ value of 453 mg/L TC was reported, and the same authors reported an EC_50_ > 1000 mg/L for sulfamethoxazole [24]. For EN quite high toxicity values were determined in the present work. Migliore et al. [25] determined a significant change in root growth already at a concentration of 5 mg/L EN for four different crops (*Cucumis sativus* L., *Lactuca sativa* L., *Phaseolus vulgaris* L. and *Raphanus sativus* L.). In the current experiment, a root growth reduction of 25% was observed at 55.4 mg/L EN. Migliore et al. [25] germinated the seeds prior to exposing them to EN-containing nutrient medium and cultivated the seedlings under light and over a longer duration of time. The differences in sensitivity are most likely attributed to these differences in the experimental setup. The results indicate that EN unfolded a stronger effect on young seedlings than at a very early stage in root development. Moreover, Migliore et al. [25] provided no details on the pH of the medium. Pre-trials of the current study revealed that the pH is of high importance for plant response in the toxicity studies. In the here performed phytotoxkits a pH of 6 in 0.5 g/L citric acid buffer was proven to be suitable to dissolve all test compounds and at the same time, did not affect the seed germination by self.

Therefore, it is obvious that very different toxicity values can be achieved depending on the experimental conditions (duration of the experiment, light conditions, pH, medium) and the investigated crop and its growth stage for a given toxic compound.

One major disadvantage of the phytotoxkits is that only compounds can be analyzed that are soluble in the chosen buffer. Precipitation of compounds during the test can result in erroneous conclusions, which will most likely underrate the toxicity of the compound. Another disadvantage is the short duration of the experimental setup of 120 h, as especially elements like copper seem to unfold a toxic effect only over longer exposure time.

The results of the current study indicate that combinatory effects take place when more than one antibiotic is available. The EC of antibiotics seems to decrease when more than one antibiotic exists in the soil system or enter the soil via organic amendments. In contrast, it was shown by Riaz et al. [26] in a short-term toxicity experiment with wheat (*Triticum aestivum* L.) that a mixture of fluoroquinolone antibiotics acted antagonistically in their effect on biomass development. In the present experiment, all three classes of antibiotics showed a synergistic effect on root growth when two antibiotics from different classes were available at significant concentrations at the same time.

### 3.2. Is It Possible to Transfer the Data Obtained by Phytotoxkits to Vegetation Trials?

The main advantage of the phytotoxkits is the fast result on the toxicity of single compounds as well as on mixtures. Such results can be transferred to marine or limnic systems, but the transfer is difficult with respect to soils. Adsorption to soil organic matter, oxides, and hydroxides, and clay minerals is of high relevance and can change the toxicity of a given compound. Sulfonamides and fluoroquinolones were strongly adsorbed by soil organic matter [27], while adsorption and fixation to clay minerals are important processes, especially for fluoroquinolones and tetracyclines [28,29]. Tetracyclines and fluoroquinolones have larger soil sorption coefficients and are relatively immobile in the soil, while sulfonamides show weak soil sorption and higher mobility within the soil [30,31]. Therefore, results based on phytotoxkits give only a first indication on the toxicity of a compound and on combinatory effects of different compounds, which needs confirmation under soil conditions where adsorption, as well as degradation, can occur.

In the current study, a pot trial was conducted to prove the relevance of the phytotoxkit results on plant growth under greenhouse conditions. The sandy substrate of the trial still has to be seen as an artificial setup but delivers the possibility to study the toxic effects over a longer period of time where plants can complete their lifecycle when all necessary nutrients and water were supplied in sufficient amounts. Moreover, root architecture is closer to natural conditions in a substrate trial as plants react very different in hydroponics compared to soil culture experiments. Redjala et al. [32] observed that the *Casparian strip* is not perfectly formed at the root tips of plants grown in hydroponics so that solutes are not retained but can directly enter into the xylem. The phytotoxkits are not comparable to hydroponics as the seeds were placed on wet filter paper but were not soaked in the nutrient solution. Nevertheless, it can be expected that the root architecture is also artificial under the conditions of the phytotoxkit.

The most significant difference in the soil experiment was the very strong sorption of EN to the substrate. Only traces of EN could be detected in soil solution (suction water), and fractionation of the soil revealed that most of the EN, which could be extracted, was bond to the soil surface (Figure 7). Despite of the strong sorption, traces of EN were taken up by the plants and were transferred to the vegetative plant parts. The data in Table 5 revealed that EN was taken up by the plant in a dose-dependent manner, but the translocation factor was lower at the very high EN dose of 50 mg/pot, where also the biomass production was negatively affected by the high antibiotic concentration (Figure 5). Also, other antibiotics were detected in plants and were taken up in a dose-dependent manner [see review 33]. Very different effects were summarized in the review of Tasho and Cho [33], but very often, affects on root growth were reported and changing effects for different plant organs. Chung et al. [34] determined that only trace amounts of EN and other antibiotics were taken up by radish. The uptake accounted for 0.08–3.9% of the EN that was spiked to the soil in that study. The uptake was lower in the here presented study and accounted for only 0.001–0.017% of the amount that was applied to the pots. The maximum application in the present study was three times lower than in the study from Chung et al. [34], and the EN was supplied to the top of the soil while Chung and coworkers thoroughly mixed EN with the soil.

The suction water and the soil analysis (Figure 7) revealed that nearly no transport of EN occurred in the soil, which is most likely the reason for the low EN uptake by the plants. A quite high uptake of EN of 9.2–16.9 µg/kg FW was reported for red cabbage by Chowdhury et al. [35] from a field experiment where pig slurry was applied spiked with 150 mg/kg EN. The EN contents in the leaves of *Sinapis alba* L. in the current experiment were in a comparable range, when calculated on a fresh weight basis and accounted for 1.5–31.5 mg/kg FW. It can be assumed that plants grown in natural soils will take up only low proportions of the EN that is applied to the soil because of the strong adsorption. Nevertheless, antibiotics can be detected in the edible plant parts, as shown by Chowdhury et al. [35] as well, and can enter the food chain this way.

The phytotoxkits indicate to a hormetic effect of TC and SD at subinhibitory concentrations (TC: 18.8 mg/L and SD: 2.3 mg/L), which was not observed for EN at the investigated concentrations (Figure 2). Hormetic effects were also reported in the literature for sulfonamides when applied at lower doses [36] and for tetracyclines [37].

For the phytotoxkit an EC_50_ value of 265 mg/L EN was determined. Root growth in the pot trial was reduced by 50% at a calculated concentration of 46.6 mg/pot, which would correspond in maximum to approximately 50 mg/L soil solution assuming that the total amount of EN was in solution, which was not the case as indicated by the suction water samples. Therefore, the EC_50_ value seems to be lower for EN, when the plants were grown in pot trials over a longer growth period, what was also indicated by the study of Migliore et al. [25]. For TC, the opposite was reported. Here lower inhibitory effects of TC were detected in the soil than in solution, which was explained by the strong adsorption of TC onto soil components (clay and organic matter) [30,38,39,40].

The results clearly reveal that it is not possible to transfer phytotoxkit results directly to soil systems but that they give a good indication about toxicity in general and the magnitude of the range of toxicity. To get further inside into the toxicity in the soil-plant system, it is necessary to perform tests, which are closer to natural systems or build up the processes that are relevant for the toxic reaction.

### 3.3. Copper in the Plant Toxicity Test

Cu is an element needed by the plants because of its essential function as an enzymatic cofactor in several redox-reactions and constituent of important molecules. Excess Cu is toxic for plants because in its ionic form, Cu is highly reactive and can bind to histidine or sulfhydryl groups and by this in-activate the functions of proteins [41]. Moreover, Cu can cause oxidative stress by reacting with small molecules such as O_2_, causing the release of reactive oxygen species. Oxidative stress can lead to the destruction of proteins and membrane structures [42]. Therefore, plants need a tight Cu homeostasis to guarantee the uptake and transport of sufficient amounts of Cu to target organs and organelles but to prevent, on the other side, the accumulation of excess and thereby toxic Cu concentrations [43]. Cu is bond strongly to the soil matrix and is especially complexed by the soil organic matter and by Mn- and Fe-oxides and hydroxides, and only a very low proportion is generally available [44]. The toxicity tests with Cu have to be seen in this context.

Cu was applied in the pot trial with a maximum amount of 6 mg/pot. Cu applications of 1.7–2.5 mg/L were reported to reduce root growth by 50% [45]. Increasing Cu application revealed no negative effects on biomass development of *Sinapis alba* L. in the here presented trial, but the Cu content increased in all plant parts in a dose-dependent manner (Table 4). At least the statistics revealed a combinatory effect of Cu and EN on biomass development of the roots. With increasing Cu concentration, the toxicity of EN seemed to increase in that way that less root biomass was build (Table 3). Graouer–Bacart et al. [46] could show that Cu(II) and EN interacted at the water-soil interface, and the proportion of adsorbed EN increased in the presence of Cu as co-adsorption was observed. The formation of Cu-EN-complexes can change the environmental behavior of both contaminants, which was investigated for other heavy metals as well. For example, in the presence of EN, increased Cd bioaccumulation and, therefore, enhanced Cd toxicity was determined for earthworms [11]. The presence of Cu resulted in a different bio-concentration of EN in zebrafishes [47]. Sayen et al. [48] showed that the Cu uptake by *Phragmites australis* (Cav.) was slightly reduced when EN was available at the same time because of the formation of Cu-EN complexes. Moreover, the proportion of free EN was reduced in the presence of Cu due to complexation in that study. In the present study, no significant effect of EN on Cu uptake of *Sinapis alba* L. could be observed (Table 4) only on root biomass production.

## 4. Materials and Methods

### 4.1. Toxkits

Toxicity tests purchased from MicroBioTests Inc. (Gent, Belgien) were used in the present study. Such tests are suitable to observe the short-term effect of soluble xenobiotics on early plant growth. Testkits guarantee good reproducibility and comparability as they can be conducted repeatedly under the same conditions. With phytotoxkits the effect of single compounds on germination and root development was studied. The disadvantage of this phytotoxkit is the strong restriction in experimental time, which is limited to 120 h. *Sinapis alba* L. was chosen as a test crop because root development revealed big differences in relation to the applied antibiotics, and the seedlings showed a relative even root growth. Moreover, *Sinapis alba* L. is a plant of agronomic relevance that was later on used in the pot trial as well.

Several factors strongly affect the results of toxicity test such as the pH of the test solution or EDTA addition, which change the bioavailability of certain compounds. It is of major importance to control the pH during the test procedure as the test compounds can have an effect on pH, which can counteract or fortify the result of the whole test system [5]. Therefore, in a first step, the buffering system of the phytotoxkit was adjusted in that way that a stable pH was achieved and that all test compounds could be solved under the same conditions. Moreover, it is of major importance that the chosen buffer system does not affect seed germination by self, because that could affect the results as well. The compounds tested by phytotoxkits were analytical standards of the antibiotics enrofloxacin (EN, purity 99.2%), tetracycline (TC, purity 98.0%) and sulfadiazine (SD, purity 99.3%) all purchased from Sigma-Aldrich and copper applied in the form of CuCl_2_. Different buffer systems were compared (TRIS buffer at pH 7.8 and 8.1, Mc Ilvaine buffer at pH 6 and 7.8 and citric acid buffer at pH 6) to select a buffer system suitable for the solution of the different antibiotics and copper. A citric acid buffer (0.5 g/L citric acid) at pH 6 was found to be suitable to dissolve all test-compounds, and the buffer did not negatively affect root development by self. Therefore, this buffer system was used in all seed germination toxicity tests.

A phytotoxkit consists of a plastic box, a sponge, which take up and store the test solution together with a thick white and a thin black filter paper where 10 seeds were placed on. The sponge and filter paper were placed in the plastic box, and 20 mL of test compound dissolved in citric acid buffer was distributed to the sponge. The black filter was placed on the wet white filter paper, and the seeds were placed in an even row on the paper. Then the box was closed and was put in the dark for 120 h at 23 °C. Afterward, photos were made from the seedlings (see Figure 1), and the root length was determined by the software Inkscape that was provided with the toxkits by MicroBioTests Inc. (Gent, Belgien).

The concentrations of the antibiotics and copper that were tested in the phytotoxkit are summarized in Table 6.

### 4.2. Effect of Increasing Concentrations of Enrofloxacin and Copper on Plant Growth of Sinapis alba L.

The toxicity of EN and Cu was tested in a pot experiment using Mitscherlich containers filled with 7 kg of washed sand. All necessary nutrients were applied in sufficient amounts as solutions prepared from pure chemicals to the sand before seeds were sown, and EN and Cu were applied.

The pot experiment was conducted in an open cage greenhouse under ambient light and temperature conditions. EN and CuCl_2_ were diluted in citric acid buffer (0.5 g/L citric acid) at a pH of 5.5. Cu application diluted in 100 mL was applied six days before sowing, and EN dissolved in 100 mL 2 days before sowing. Cu and EN were administered at the following doses:

EN: 0–5–25–50 mg/pot containing 7 kg of washed sand

Cu: 0–1–3–6 mg/pot containing 7 kg of washed sand

Cu was also part of the homogenous fertilization with micronutrients so that all pots received additionally 0.2 mg Cu/pot as it was not the target of the trial to induce Cu deficiency. For the sake of convenience, this is not mentioned in the tables and figures.

The trial was arranged in a completely randomized design with four repetitions per treatment and with all possible combinations of Cu and EN application. Plants (nine seeds per pot) were sown on 9 June 2016, and were harvested 28 days later at the beginning of the flowering stage. The vegetative biomass was divided into stems and leaves, and the fresh weight was determined. Roots were harvested and were washed carefully with distilled water to remove adhering sand and were dried by paper towels before the determination of the fresh weight.

All plant samples were frozen in liquid nitrogen and afterward freeze-dried (Gamma, Christ, Osterode, Germany). The dry weight was recorded for the determination of the dry matter yield. For further analysis, the freeze-dried plant material was ground to a fine powder (particle size of <60 µm) by using vibrating disc mill (RS1 Retsch, Haan, Germany) with zirconium oxide grinding equipment to prevent metal contaminations of the samples during grinding.

From each treatment additionally, one pot was prepared without vegetation. In these pots, suction tubes were installed 5 cm below the surface. Suction water samples of 2 mL were taken regularly every five days. At the end of the trial, core soil samples were taken from these pots by using an auger and were divided into different layers: 0–4 cm, 4–8 cm, and 8–12 cm. In addition, a surface sample (0–1 cm) was taken.

### 4.3. Determination of Copper in Plant Material

0.5 g fine-ground plant material was digested with 6 mL HNO_3_ (supra 69%) + 1.5 mL H_2_O_2_ (30%) in a microwave oven (CEM/Mars). Samples were heated at 800 W in 5 min to 120 °C and temperature was held for 2 min; then the vessels were heated at 800 W to 200 °C in 10 min, and the temperature was hold for 15 min; afterward, samples were cooled down at 400 W to 50 °C within 30 min, and the temperature was held for 5 min. After cooling down, the digest was filled up to 50 mL with de-ionized water. The Cu concentration was determined by ICP-MS (Element XR, Thermo, Dreieich, Germany).

### 4.4. Determination of Enrofloxacin in Plant Material, Suction Water and Substrate

0.75 g fine-ground, freeze-dried plant material was weight in falcon tubes, and an internal standard was added (50 µL of a standard mixture containing 10,000 µg/L of norfloxacin and ofloxacin each in methanol). Afterward, the plant material was extracted with 20 mL 0.2 M citric acid buffer (pH 3.7) containing Na_2_-EDTA (Di-Sodium-Ethylendiamintetraessigsäure) for 20 min in an ultrasonic bath followed by a centrifugation step at 10,000× *g* for 6.5 min at room temperature (RT). The supernatant was put in a conical flask, and the pellet was extracted again with 20 mL 0.2 M citric acid buffer (pH 3.7) containing Na_2_-EDTA as described before. Supernatants were combined, and the pellet was extracted for a third time by using 10 mL methanol (80%) containing 20% of citric acid buffer (pH 3.7). The extraction procedure was the same as described before, but the supernatant was transferred in an extra falcon tube. The pellet was mixed with 10 mL methanol containing 1% formic acid and was incubated over-night at room temperature. The sample was centrifuged again the next day, and the methanolic supernatants were combined and concentrated to 5 mL by using a vacuum concentrator (Eppendorf, Hamburg, Germany). This fraction was combined with the aqueous fractions in the conical flasks and different cleaning procedures were conducted. First, a liquid/liquid extraction with 10 mL of heptane was conducted, and the aqueous fraction was collected, filled up to 100 mL, and adjusted to pH 3 by adding formic acid. The extract was vacuum-filtrated through filter paper (Macherey-Nagel 85/90 BF) before a further cleaning step through SPE cartridges (Phenomenex Strata-X, Aschaffenburg, Germany) was performed. SPE cartridges were eluted with 3 mL methanol and 3 mL citric acid buffer (pH3) for conditioning of the cartridges before the samples were loaded on the SPE cartridge at a flow rate of 5 mL/minute. Afterward, the cartridges were washes with 8 mL of water and were dried under vacuum. For elution of the antibiotics, the cartridge was first eluted with 4 mL of methanol and in a second step with 4 mL of methanol containing 1% formic acid. The eluate was concentrated to 1 mL and then quantitatively transferred into a 2 mL graduated flask and filled up with methanol. Afterwards the sample was filtrated through a syringe filter holder (Wicom perfect flow, PVDF, 0.2 µm) into a vial. The measurement was conducted using a liquid chromatography mass spectrometry (LC-MS/MS) system (AB Sciex 4000 Q-Trap, Darmstadt, Germany), and an XB-C18 column (Kinetex 2.6µ XB-C 18 100A column 100 × 2.1 mm, Phenomenex, Aschaffenburg, Germany). Eluent (A) was water containing 77.08 mg/L ammonium acetate and 0.1% formic acid and eluent (B) methanol containing 0.1% formic acid. A gradient was used starting with 83% A, which decreased to 65% A within 13 min and further on to 8% A in the next 3 min. 8% A was held for 4 min and then the proportion of A was raised to 83% again within 1 min and held at 83% for further 8 min. Quantification of enrofloxacin was performed by a standard addition procedure.

Determination of enrofloxacin in suction water and sand: 2 mL of suction water was first filtrated through a syringe filter holder (Wicom perfect flow, PVDF, 0.2 µm) and was then concentrated by using a concentrator (Eppendorf, Hamburg, Germany). The dry pellet was resuspended in 100 µL methanol containing 1% formic acid using an ultrasonic bath for 5 min. Afterward, the sample was centrifuged at 15,000 rpm for 1 min, and 50 µL of the supernatant was filled in vials and measured by LC-MS/MS for EN as described in Section 4.4. Only at the first sampling traces of EN could be determined, which were below the limit of quantification.

The sand was dried at 45 °C before 15 g sand was extracted with 40 mL methanol containing 1% formic acid in an ultrasonic bath for 20 min. The supernatant was filtrated, concentrated in the vacuum concentrator, and filled up to 2 mL with methanol. This volume was filtrated through a syringe filter holder (Wicom perfect flow, PVDF, 0.2 µm) into a vial and was determined by the same procedure as described before at the LC-MS/MS.

### 4.5. Statistics

The phytotoxkit results were used to evaluate different effect concentrations (EC values). The observed root length was plotted against the tested concentrations, and EC values were calculated by logarithmic regressions and effect concentrations such as EC_25_, EC_50_, or EC_70_ were obtained.

The translocation factor (Tf) was calculated using the following equation according to Zacchini et al. [49], which calculate the percentage of a contaminant that is transferred from the roots to the aerial (stems + leaves) plant parts.

$$Tf=\frac{\mathrm{Contaminant}\;\mathrm{concentration}\;\mathrm{in}\;\mathrm{aerial}\;\mathrm{plant}\;\mathrm{parts}\;\left(µg/\mathrm{plant}\right)}{\mathrm{Contaminant}\;\mathrm{concentration}\;\mathrm{in}\;\mathrm{the}\;\mathrm{roots}\;\left(µg/\mathrm{plant}\right)}\times 100$$

Statistical data analysis was conducted using the COSTAT software package, employing the analysis of variance (ANOVA) and by Tukey’s test. Significant differences were determined at *p* < 0.05. A two-factorial ANOVA was performed to separate the effect of Cu and EN application.

## 5. Conclusions

Phytotoxkits are a useful tool to get a first indication of the toxicity of a compound on early plant growth. For aquatic systems, toxkits can deliver valuable information as results can be directly transferred to ecosystems because the medium is water in the test system and in the environment, too. The missing solubility of some test compounds can be seen as a major disadvantage of phytotoxkits, as the precipitation of compounds can cause erroneous values and can lead to wrong conclusions. Moreover, the pH is of paramount importance as toxicity characteristics can change with pH. Many compounds, such as antibiotics, show a direct effect on pH and can affect plant growth by this and the results obtained by the phytotoxkit.

The short experimental duration of the phytotoxkits is another restriction of the method because many antibiotics show no acute toxicity but can show long-term effects on reproduction.

The combinatory effect of Cu and EN was studied on the growth performance of *Sinapis alba* L. exemplary for terrestrial plants. The results show that besides pH also the medium and the exposure time are of high relevance for plant growth. It is not possible to transfer EC-values, which were derived from phytotoxkits to soil conditions or plant development. Most antibiotics show adsorption to the soil matrix or to the soil organic matter, and this can change bioaccumulation and the toxicity considerably. Adsorption of EN to soil organic matter as proposed by Teixido et al. [50] can be neglected in the presented pot trial as sand was used as a substrate. Therefore, in our case, adsorption resulting from charge-charge interactions between EN and quartz sand surfaces as observed by Hari et al. [51] seemed to be likely, as EN was strongly bound to the soil surface and could not be detected deeper than 5 cm below the surface. This indicates a complex situation under field conditions, where the toxicity of a compound is determined by many other factors like soil characteristics (pH, organic matter, soil texture, and soil structure), plant species, growth stage, and climatic conditions.

Nevertheless, the trials revealed that direct toxicity on plant growth could only be expected when very high concentrations of antibiotics were applied to agricultural fields with highly contaminated organic nutrient sources. Nevertheless, sometimes quite high contaminations were reported in literature like for pig slurry with a sulfadiazine content of up to 235 mg/L FW and a tetracycline content up to 66 mg/L FW [52], which could have a direct negative effect on plant germination and root development. Especially, when more than one antibiotic is available in an organic fertilizer or when additionally, heavy metals are constituents, combinatory effects can be expected that can enhance the toxicity toward plants. Further studies are necessary, investigating a combinatory effects of different antibiotics and in combination with heavy metals to get a deeper insight into the complex nature of the toxicity of organic nutrient sources. Moreover, toxicity tests should be improved, which better built up the toxicity at the soil-plant interface.

## Figures and Tables

**Table 1:** Determination of effect concentrations (EC_25_, EC_50_, and EC_70_) for different antibiotics and copper on the root growth of *Sinapis alba* L. using a commercial phytotoxkit (MicroBioTests Inc. Gent, Belgium; tests performed in 0.5 g/L citric acid buffer at pH 6).

Test Compound		Effect Concentrations [mg/L]		
Antibiotic	Metal	EC_25,120h_	EC_50,120h_	EC_70,120h_
Enrofloxacin (EN)		55	265	926
Tetracycline (TC)		40	109	241
Sulfadiazine (SD)		2.7	9.8	28
	Copper (Cu)	No effect at 0.5–24 mg Cu/L during the test duration of 120 h		

Effect concentrations were recorded at variable concentrations: 2.34–4.69–9.38–18.75–37.5–75–150 mg/L for SD; 18.75–37.5–75–150–300 mg/L for EN, and 18.75–37.5–75–150–300 mg/L for TC; 0.5–1–2–4–6–12–24 mg/L for Cu.

**Table 2:** Combinatory toxicity of enrofloxacin (EN), tetracycline (TC), and sulfadiazine (SD) on the root growth of *Sinapis alba* L. tested by phytotoxkits (MicroBioTests Inc. Gent, Belgien performed in 0.5 g/L citric acid buffer at pH 6).

Test Compound		Observed Concentration of Toxicity
Variable Concentration *	Fixed Concentration	[mg/L] EC_70_ after 120 h
EN	-	926
	SD-3 mg/L	167
	TC-30 mg/L	157
TC	-	241
	SD-3 mg/L	53
	EN-150 mg/L	142
SD	-	28
	TC-30 mg/L	6.1
	EN-150 mg/L	0.8

* variable concentrations: 2.34–4.69–9.38–18.75–37.5–75–150 mg/L SD, or 18.75–37.5–75–150–300 mg/L EN or 18.75–37.5–75–150–300 mg/L TC.

**Table 3:** Root growth and vegetative growth of *Sinapis alba* L. 28 days after sowing in relation to copper and enrofloxacin application (two-factorial ANOVA).

Factors	Roots [g FW/pot] [g DW/pot]		Vegetative Biomass [g FW/pot] [g DW/pot]	
Copper [mg/pot]				
0	39.9 a	4.7 a	83.4 a	8.0 a
1	37.2 a	4.6 a	75.2 a	7.0 a
3	36.8 a	4.8 a	81.6 a	7.8 a
6	33.6 a	4.1 a	81.6 a	8.1 a
Enrofloxacin [mg/pot]				
0	46.1 a	5.7 a	90.2 a	8.6 a
5	42.7 ab	5.8 a	93.1 a	9.2 a
25	37.4 b	4.7 a	85.3 a	8.2 a
50	20.4 c	1.9 b	53.2 b	4.7 b
Interaction	**	ns	ns	ns

Means followed by different letters indicate statistical differences in comparison to the control using Tukey’s test at *p* ≤ 0.05.

**Table 4:** The copper content in leaves, stems, and roots of 28-day old *Sinapis alba* L. plants in relation to copper and enrofloxacin application to the pots (two-factorial ANOVA).

Factors	Leaf [mg Cu/kg DW]		Stem [mg Cu/kg DW]		Root [mg Cu/kg DW]	
Copper [mg/pot]						
0	7.1	c	4.2	c	13.2	c
1	8.6	c	4.9	bc	14.9	c
3	12.7	b	5.6	b	21.8	b
6	17.7	a	6.8	a	33.7	a
Enrofloxacin [mg/pot]						
0	12.2	a	5.3	ab	20.4	a
5	12.5	a	5.3	ab	20.1	a
25	10.9	a	5.0	b	20.6	a
50	10.5	a	5.9	a	22.4	a
Interaction	ns		ns		ns	

Means followed by different letters indicate to statistical differences in comparison to the control using Tukey’s test at *p* ≤ 0.05.

**Table 5:** Enrofloxacin content (±standard deviation) in leaves, stems, and roots of *Sinapis alba* L. plants 28 days after sowing in relation to enrofloxacin application and calculated translocation factors (Tf) from roots to aerial plant parts.

Enrofloxacin [mg/pot]	Leaf [µg EN/kg DW]	Stem [µg EN/kg DW]	Root [µg EN/kg DW]	Tf [%]
0	nd	nd	nd	nd
5	17.0 ± 4.8	19.7 ± 4.6	46.3 ± 21.2	69
25	46.2 ± 27.9	29.4 ± 17.5	172.7 ± 179.5	83
50	115.7 ± 133.7	123.1 ± 157.4	480.9 ± 346.2	51

Tf = translocation factor: percentage of accumulated EN that was transferred into the aerial plant parts (leaves and stems) in relation to that remaining in roots calculated as uptake on the whole plant basis; nd = not determined.

**Table 6:** Concentrations of antibiotics and copper that were tested as single compounds or in combination using a phytotoxkit (MicroBioTests Inc. Gent, Belgium; plant growth tests performed in 0.5 g/L citric acid buffer at pH 6).

Test Compound	Fixed Concentration [mg/L]	Test Compound	Variable Concentration [mg/L]
**Single Compounds:**			
Enrofloxacin (EN)			18.8–37.5–75–150–300–400–500–600–700–800–1000
Tetracycline (TC)			18.8–37.5–75–150–300
Sulfadiazine (SD)			2.3–4.7–9.4–18.8–37.5–75–150
Copper (Cu)			0.5–1–2–4–6–12–24
**Combinations:**			
EN	150	+TC	18.8–37.5–75–150–300
EN	150	+SD	2.3–4.7–9.4–18.8–37.5–75–150
TC	30	+EN	18.8–37.5–75–150–300
TC	30	+SD	2.3–4.7–9.4–18.8–37.5–75–150
SD	3	+TC	18.8–37.5–75–150–300
SD	3	+EN	18.8–37.5–75–150–300
